# Genetic Diversity and Genetic Structure of the Wild Tsushima Leopard Cat from Genome-Wide Analysis

**DOI:** 10.3390/ani10081375

**Published:** 2020-08-07

**Authors:** Hideyuki Ito, Nobuyoshi Nakajima, Manabu Onuma, Miho Murayama

**Affiliations:** 1 Kyoto City Zoo, Kyoto 606-8333, Japan; 2 Wildlife Research Center, Kyoto University, Kyoto 606-8203, Japan; mmurayama@wrc.kyoto-u.ac.jp; 3 Wildlife Genome Collaborative Research Group, National Institute for Environmental Studies, Tsukuba 305-8506, Japan; naka-320@nies.go.jp (N.N.); monuma@nies.go.jp (M.O.)

**Keywords:** Tsushima leopard cat, GRAS-Di, genetic diversity, genome-wide analysis

## Abstract

**Simple Summary:**

The Tsushima leopard cat, *Prionailurus bengalensis euptilurus*, is a small regional population of the Amur leopard cat and is only found on Tsushima Island in Japan. A breeding program will require adequate information on parentage, kinship, and inbreeding for this population. Hence, there is an urgent need to develop this information in order to conserve the population and its genetic diversity. We performed GRAS-Di analysis to investigate the genetic diversity and genetic structure of the Tsushima leopard cat. We identified between 133 and 158 single-nucleotide polymorphism (SNP) markers in three different genotyping methods. These SNP markers can be used in identification of individuals and parentage. In addition, structure analysis using these markers demonstrated the similar genetic composition of the samples from 48 Tsushima leopard cats, and indicated Tsushima leopard cats have no subpopulations. We have provided genetic markers that are useful for conservation of the Tsushima leopard cat, such as individual identification and parentage. Moreover, we have also clarified units for conservation of the Tsushima leopard cat population from structure analysis.

**Abstract:**

The Tsushima leopard cat (*Prionailurus bengalensis euptilurus*) lives on Tsushima Island in Japan and is a regional population of the Amur leopard cat; it is threatened with extinction. Its genetic management is important because of the small population. We used genotyping by random amplicon sequencing-direct (GRAS-Di) to develop a draft genome and explore single-nucleotide polymorphism (SNP) markers. The SNPs were analyzed using three genotyping methods (mapping de novo, to the Tsushima leopard cat draft genome, and to the domestic cat genome). We examined the genetic diversity and genetic structure of the Tsushima leopard cat. The genome size was approximately 2.435 Gb. The number of SNPs identified was 133–158. The power of these markers was sufficient for individual and parentage identifications. These SNPs can provide useful information about the life of the Tsushima leopard cat and the pairings and for the introduction of founders to conserve genetic diversity with ex situ conservation. We identified that there are no subpopulations of the Tsushima leopard cat. The identifying units will allow for a concentration of efforts for conservation. SNPs can be applied to the analysis of the leopard cat in other regions, making them useful for comparisons among populations and conservation in other small populations.

## 1. Introduction

**Figure 1:**
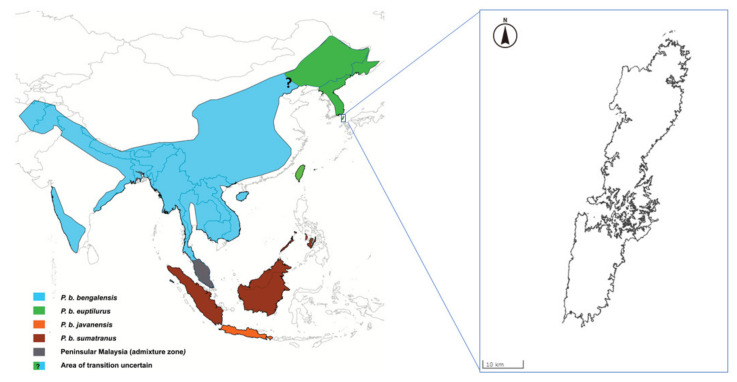
Distribution of the leopard cat subspecies (adapted from Patel et al. (2017) [3]) and location of Tsushima Island (modified map from the Geospatial Information Authority of Japan; https://maps.gsi.go.jp/).

The leopard cat (*Prionailurus bengalensis)* is widely distributed throughout South Asia, East Asia, and Southeast Asia. The Leopard cat is classified as Least Concern by The International Union for Conservation of Nature (IUCN) Red List of Threatened Species [1]. The leopard cat is generally classified into 12 subspecies [2]. A recent genetic study suggested that the leopard cat has only four subspecies [3]. The Amur leopard cat (*Prionailurus bengalensis euptilurus*), which is one of the subspecies, is distributed in Far East Asia. The Tsushima leopard cat is found only in Tsushima, Japan (Figure 1) and was classified as a regional population of the Amur leopard cat in recent genetic study [3]. Wild Tsushima leopard cat populations are declining due to habitat fragmentation, depletion of feeding grounds, and deaths due to impacts with vehicles. The population in the wild is estimated to be less than 100 animals [4]. Therefore, the Tsushima leopard cat has been awarded National Nature Monument status and has been classified as a critically endangered species on the Japanese Red List. In 1995, the Japanese Ministry of the Environment established a protection and breeding program for this wild cat.

The conservation of endangered species relies on detailed knowledge about nutrition, reproduction, husbandry, veterinary medicine, genetics, and more. Genetic information is especially important for small populations because they are susceptible to various genetic influences, including genetic drift, accumulation of deletion traits, and loss of genetic variations. The population of the Tsushima leopard cat is small, so a conservation strategy for this animal based on genetic information is important. Several genetic studies (e.g., mitochondrial DNA, Y chromosome DNA sequences, and microsatellite markers) have been performed, and the results have indicated that the Tsushima leopard cat has less genetic variation than other Asian leopard cat populations [5,6,7]. Generally, a captive breeding program can be established through genetic monitoring, usually requiring accurate parentage determination and estimation of individual kinship and inbreeding [8]. However, the identification of individuals in the Tsushima leopard population is difficult using microsatellite marker analysis due to the population’s low genetic diversity [7], so more DNA markers must be developed to design an appropriate conservation program.

Microsatellite markers have many advantages, which have resulted in their wide use in the field of molecular ecology [9,10,11]. Single-nucleotide polymorphisms (SNPs) are enriched and relatively uniformly distributed throughout the genome, making them valuable genetic markers as well as microsatellites. Recently, advantages such as ease of genotyping, genetic stability, and suitability for automation have increased the use of SNPs [11,12,13]. Further, the advance of molecular genetics technology through next-generation sequencing (NGS) has made it possible to obtain extensive nucleotide sequences, enabling rapid and large-scale development of DNA markers. Moreover, more economical and efficient approaches using NGS have been developed, such as restriction site-associated DNA sequencing (RADSeq) [14], double digest RADSeq (ddRADSeq) [15], MIG-Seq (multiplexed ISSR genotyping by sequencing) [16], genotyping-in-thousands by sequencing (GT-Seq) [17,18], and genotyping by random amplicon sequencing, direct (GRAS-Di) [19,20]. These approaches enable simultaneous sequencing and genotyping of thousands of SNP markers, and they have been used in various species, including endangered species [18,21]. Although GRAS-Di has few applications thus far, GRAS-Di is useful in characterizing wildlife because it can function with the available low-quality DNA in a polymerase chain reaction (PCR)-based method and does not require a reference genome, similar to MIG-seq [19,20]. GRAS-Di is a technology that produces a sequence library by two PCRs using random primers and adapter sequences, and detects SNPs using NGS. GRAS-Di is expected to develop more SNPs than MIG-seq because it is a non-targeting method using short random primers, which is an advantage of this approach. The purpose of this study was to identify SNPs using genome-wide analysis with the GRAS-Di approach that would clarify the genetic diversity and structure of the wild Tsushima leopard cat.

## 2. Materials and Methods

### 2.1. Sample Collection

This study was conducted in strict accordance with the guidelines for ethics in animal research established by the Wildlife Research Center of Kyoto University. The Kyoto City Zoo approved the research (#20170426). Muscle samples were obtained from 2004 to 2015 from 48 Tsushima leopard cats that had been either killed in road accidents or rescued from road accidents and later died. All muscle samples were stored in ethanol until DNA extraction using the QIAGEN DNeasy Blood and Tissue Kit (Qiagen, Hilden, Germany). DNA concentrations were measured using a NanoDrop spectrophotometer (Thermo Fisher Scientific, Waltham, MA, USA).

### 2.2. Construction of the Draft Genome

The isolated genomic DNA was used to construct short-insert libraries using TruSeq DNA PCR Free (350) (Illumina, San Diego, CA, USA), following standard protocols provided by Illumina. This was followed by 150-bp paired-end sequencing using a whole-genome shotgun sequencing strategy on the Illumina Hiseq X Ten platform. Library construction and sequencing took place at Macrogen Japan (Kyoto, Japan). Development of the draft genome used Genomic Workbench v. 11.01 (Qiagen). “Trim reads” were performed on all the read data. Quality trimming was set at *p* = 0.05 and the read-through adapters were removed. Reads were mapped to the cat genome using “Map reads to reference” in the global alignment mode. Default values were used for the similarity fraction (0.8) and length fraction (0.5) as parameters. Next, those portions of coverage connecting 10 or more reads by mapping were defined as contigs. Furthermore, the “De novo assemble tool” was applied using the trimmed read data in the “Create simple contig sequence” mode. Word size was 60. The process merged scaffolds and contigs using the “Join contig tool”.

### 2.3. GRAS-Di Analysis

GRAS-Di was developed by the Toyota Motor Corporation (Aichi, Japan) (patent ID P2018042548A) [20,22]. Library preparation and sequencing were done by Bioengineering Lab. Co., Ltd., under a license agreement. The library was constructed using two-step tailed polymerase chain reaction (PCR). The first group of PCR primers included Illumina Nextera adaptor sequences plus three-base random oligomers, and the second group of PCR primers included the Illumina multiplexing dual index and P7/P5 adapter sequence. The first PCR was performed in a 25 µL reaction volume containing 5.0 µL of 5× PrimeStar buffer (Mg^2+^ plus) (Takara) (Shiga, Japan), 2.0 µL of dNTP mixture (2.5 mM of each), 10 µL of random primer mix (100 µM), 0.25 µL of PrimeStar HS DNA polymerase (2.5 U/µL) (Takara), 1 µL of genome DNA (15 ng/µL), and 6.75 µL of sterile water. The reaction conditions were initial denaturation at 98 °C for 2 min and 30 cycles of 98 °C for 10 s, 50 °C for 15 s, and 72 °C for 20 s. The second PCR was performed in a 50 µL reaction volume containing 10 µL of 5× PrimeStar buffer (Mg^2+^ plus), 4.0 µL of dNTP mixture (2.5 mM of each), 1.25 µL of P7 primer with 8 base index (10 µM), 1.25 µL of P5 primer with 8 base index (10 µM), 0.5 µL of PrimeStar HS DNA polymerase (2.5 U/µL), 1.5 µL of PCR product from the first PCR, and 31.5 µL of sterile water. The reaction conditions were initial denaturation at 95 °C for 2 min, 25 cycles of 98 °C for 15 s, 55 °C for 15 s, and 72 °C for 20 s, followed by a final extension period at 72 °C for 1 min. The final PCR products were pooled, purified using the MiniElute PCR Purification Kit (Qiagen) following the manufacturer’s protocol, and applied for sequencing on NextSeq 500 (Illumina) using the NextSeq 500/550 Mid Output Kit v. 2.5 (paired-end, 150 cycles) (Illumina).

Low-quality reads were trimmed using Sickle v. 1.33 [23]. The Nextera adaptor and other Illumina primer sequences were clipped with default settings. Trimmed reads shorter than 50 bp were removed. Moreover, sequences after 50 bases were deleted using fastx_trimmer in FASTX-Toolkit [24].

### 2.4. Genotyping

SNP analyses were performed with three methods using Stacks v. 2.5 [25]. Stacks is a method developed for the RAD analysis and has also been applied to PCR-based methods such as MIG-Seq and GRAS-Di [16,26]. In the first method, the *denovo_map pl* mapping protocol (hereafter referred to as *de_novo*) was used. The *denovo_map.pl* included six core components: building loci (*ustacks*), creating a catalog of loci (*cstacks*), and matching samples back against the catalog (*sstacks*), transposing the data (*tsv2bam*), adding paired-end reads to the analysis and calling genotypes (*gstacks*), and population genomics analysis (*populations*). In the second and third methods, the domestic cat (*Felis catus*) genome (hereafter referred to as *ref_cat*) and the Tsushima leopard cat (hereafter referred to as *ref_TLC*) draft genome were each mapped as a reference genome using the *ref_map pl* protocol, which consisted of two components (*gstacks* and *populations*). With the first method (*de_novo*), SNP calling for each individual was done using the program *denovo_map pl* of Stacks v. 2.5 with the “-m 5” option (other options set to default) to delete and discard SNPs with read depth <5. The *populations* program of Stacks v. 2.5 was used at default settings. With the other two methods (*ref_cat* and *ref_TLC*), trimmed reads were mapped onto the reference genome of the domestic cat (*Felis_catus_9.0*) and the draft genome of the Tsushima leopard cat, respectively, using BWA-mem [27]. The SAM files thus obtained were converted to BAM files and sorted using SAMtools v. 1.4 [28,29]. Any region with coverage <5 was deleted by VariantBam v. 1.4.4 [30]. SNP calling for each individual was done using the program *ref_map pl* of Stacks v. 2.5. The *populations* program of Stacks v. 2.5 was used at default settings.

### 2.5. Analysis of Genetic Diversity and Genetic Structure

PLINK v. 2.0 [31] was used to filter the dataset so that it would contain SNPs found in at least 80% of individuals (--geno 0.2) and would exclude those from individuals with more than 20% missing genotypes (--mind 0.2). As an additional quality control step, low-confidence SNPs were filtered out using PLINK v. 2.0 in terms of the Hardy–Weinberg equilibrium (*HWE*) at *p* < 0.05 and linkage disequilibrium between all possible pairs of loci. The output files from PLINK were converted to the Genepop format using PGDSpider v. 2.1.1.5 [32]. The expected heterozygosity (*He*) and observed heterozygosity (*Ho*), probability of identity (*PID*), *PID* among siblings (*PID-sib*), and probability of exclusion (*PE*) (when the other parent is known) were calculated using GenALEx v. 6.5 [33].

The SNP data were also analyzed using the program Structure in Strauto [34] using the admixture model to estimate population genetic structure. We conducted an analysis with 10 iterations for each population size (*K*) of 1 to 10, and with Markov-chain Monte Carlo (MCMC) sampling running for 500,000 generations with an initial burn-in of 300,000 generations. The *K* values described by Pritchard, et al. [35] and Evanno, et al. [36] (*LnP(K)* and Δ*K*, respectively) were then calculated to identify the most reasonable *K* using the program Structure Harvester [37] in Strauto. The *LnP(K)* is an estimate of the posterior probability of the simulation, and it is a log probability of the data at each *K*. Δ*K* is the rate of change in the log probability of data between successive *K* values.

Moreover, the most reasonable *K* values were identified using a parsimony index described by Wang (2019) [38]. Runs were averaged and visualized using CLUMPAK [39]. The population structure was also examined by carrying out principal coordinate analysis (PCoA) using GenALEx v. 6.5 [33] to visualize the genetic relationship among different individuals in two dimensions.

## 3. Results

### 3.1. Developing the Draft Genome

For development of the draft genome of the Tsushima leopard cat, 905,091,004 reads were produced, and total read bases comprised 136.7 G bp. The entire process was performed using Genomic Workbench v. 11.01. The developed assembly had a genome size of 2.435 Gb covered in 65,356 scaffolds with an average size and scaffold N50 of 37,262 bp and 152,598 bp, respectively (DDBJ Accession Number: BIMV01000001-BIMV01065356).

### 3.2. Genetic Diversity

In total, using GRAS-Di analysis, 71,634,742 raw reads with an average of 1,492,578 reads per sample were obtained for 48 individuals (DDBJ Accession Number: BIMV01000001-BIMV01065356). The three SNP data sets created using different methods resulted in specific numbers of individuals and loci (with the process for each of the three methods detailed in Table S1). After the filtering steps, the numbers of loci and individuals remaining for *ref_TLC*, *ref_cat*, and *de_novo* were 158 loci/42 individuals, 143 loci/42 individuals, and 133 loci/41 individuals, respectively.

Genetic diversity indices of the Tsushima leopard cat in three methods are summarized in Table 1 and Table 2. Average values for *Ho* and *He* in all loci for *ref_TLC*, *ref_cat*, and *de_novo* were 0.084/0.088 (0.000–0.429/0.024–0.037), 0.076/0.083 (0.000–0.405/0.024–0.410), and 0.092/0.095 (0.000–0.405/0.024–0.0354), respectively. The cumulative *PID* values for all loci for *ref_TLC*, *ref_cat*, and *de_novo* were 1.7 × 10^−11^, 1.3 × 10^−9^, and 1.9 × 10^−10^, respectively. The cumulative *PID-sib* values for all loci for *ref_TLC*, *ref_cat*, and *de_novo* were 3.1 × 10^−5^, 2.6 × 10^−4^, and 1.0 × 10^−4^, respectively. The cumulative *PE* values (when the other parent is known) for all loci for *ref_TLC*, *ref_cat*, and *de_novo* were 0.9987, 0.9965, and 0.9977, respectively.

### 3.3. Genetic Structure within the Tsushima Leopard Cat Samples

**Figure 2:**
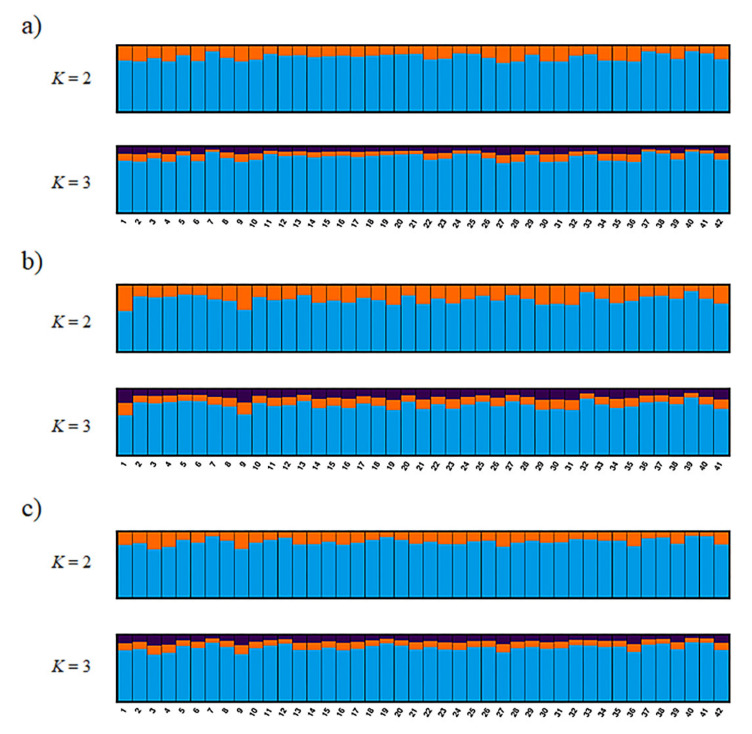
Bayesian analysis of the genetic structure in wild populations of the Tsushima leopard cat showing no deviation based on single-nucleotide polymorphism (SNP) markers. This figure was constructed by using CLUMPAK to align the 10 replicates for *K* = 2 and *K* = 3 for three genotyping methods (with all runs using the Markov-chain Monte Carlo method for 500,000 generations and an initial burn-in of 300,000 generations): (**a**) *ref_TLC*, (**b**) *ref_cat*, and (**c**) *de_novo*.

**Figure 3:**
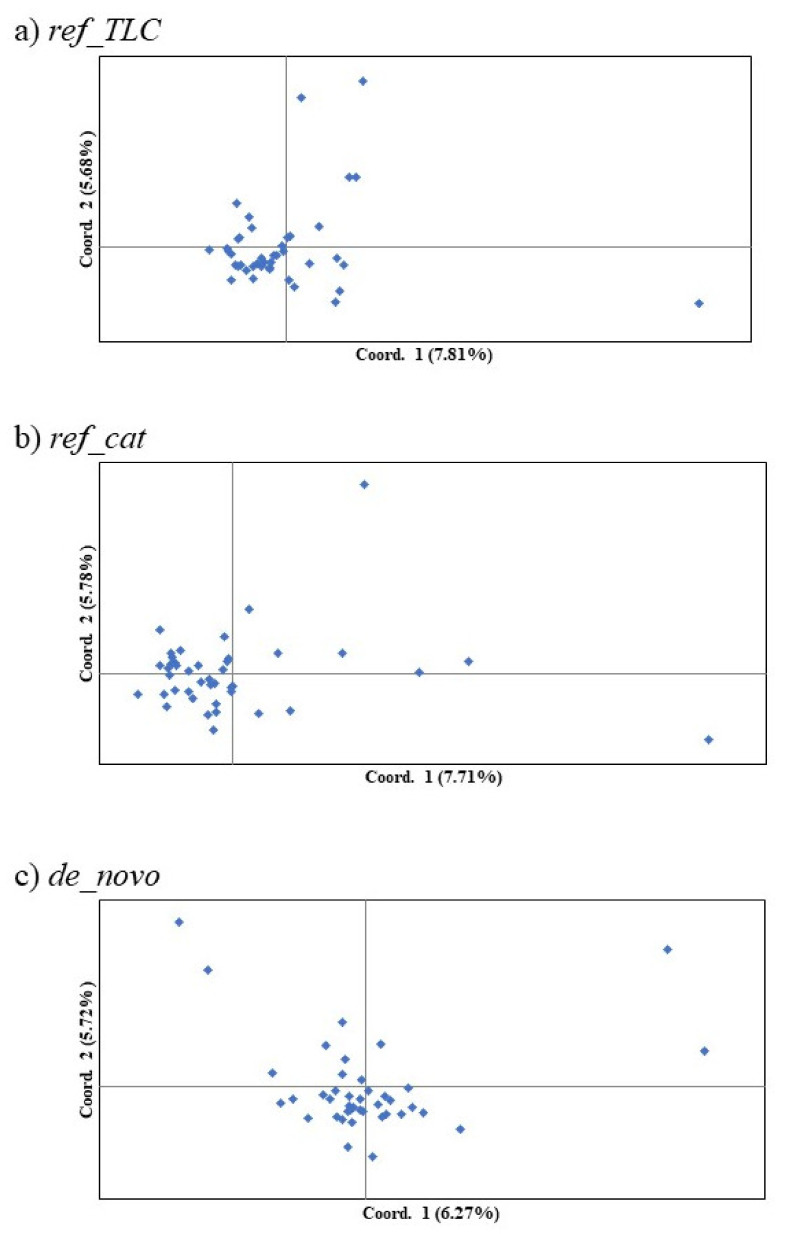
First and second components of principal coordinate analysis of SNP markers in the Tsushima leopard cat for three methods: (**a**) *ref_TLC*, (**b**) *ref_cat*, and (**c**) *de_novo*. Parentheses denote the percentages of variation explained.

The most reasonable *K* values from the three methods are displayed in Table 3. The best *K* value was *K* = 2 in all three genotyping methods for *Mean_LnP(K)*. Similarly, for parsimony, the best *K* value in all methods was *K* = 1. On the other hand, the Δ*K*, *K* = 3 in *ref_TLC* and *de_novo* and *K* = 2 in *ref_cat*, showed different values for different methods. Structure analysis and principal coordinate analysis in the three methods are demonstrated in Figure 2 and Figure 3, respectively. The bar plots of each individual for *K* = 2 and *K* = 3 with the greatest support from structure analysis are shown in Figure 2. In the PCoA, most of the individuals in all three methods were plotted in proximity to each other, with only a few individuals further away (Figure 3). The percentages of variation explained by the first two axes in *ref_TLC*, *ref_cat*, and *de_novo* were 7.81%/5.68%, 7.71%/5.78%, and 6.27%/5.72%, respectively.

## 4. Discussion

The Tsushima leopard cat is a regional population of the leopard cat in Japan, and it is a critically endangered species; several studies for conservation have been implemented [7,40,41,42]. The management of the population of the Tsushima leopard cat needs DNA markers that can not only determine individual genetic diversity but also identify individuals and clarify genetic relationships. In this study, we identified candidate SNP markers by GRAS-Di, and we compared analysis of GRAS-Di data using a de novo approach to analysis using a reference genome.

The numbers of SNP candidates detected in output data from GRAS-Di were about the same for the methods *ref_TLC* (*n* = 4859) and *ref_cat* (*n* = 4868) and less for *de_novo* (*n* = 4330). After qualitative filtering, *ref_TLC* (*n* = 158) had the highest number, followed by *ref_cat* (*n* = 143) and *de_novo* (*n* = 133). The use of a closely related species as a reference was more useful for the detection of SNP candidates, even though the reference was not of the same species. The *denovo_map.pl* analysis is expected to identify more SNP markers than the *ref_map.pl* analysis, which considers only the reads mapped to the reference genome, as all reads are considered for marker identification [43,44]. On the other hand, it was reported that there are more SNPs that are discovered by using the reference genome [45]. In addition, the reference genome has the merit of separating false positive loci from the true loci and identifying microbial DNA contaminants, and it functions as a filter [46]. Our study showed the later result. These results suggest that coverage was lower, the Tsushima leopard cat draft genome was qualitatively sufficient as a reference, there is a close relationship between domestic cats and the Tsushima leopard cat, and there was a low level of contamination in the sample DNA. The numbers of markers detected after filtering in this study were low (ranging from 133 to 158) compared with another GRAS-Di study (ranging from 4457 to 8931) [19]. Low numbers of SNPs were detected because of (i) the platform used for the analysis, (ii) the analysis pipeline, (iii) the number of individuals studied (48 samples in this study versus 59–100 samples in the previous study) [19], (iv) the number of sequenced reads per individual, (v) the genome size of the species, and (vi) the lack of genetic diversity in the Tsushima leopard cat. The various options for filtering the programs Stacks and PLINK, used for bioinformatics analysis, have been shown to significantly affect the resulting number of SNPs [47,48]. However, the values of each option are not identical across all datasets [47], making it difficult to compare the assessment of genetic diversity with the number of SNPs detected. Therefore, the low SNP numbers found in the Tsushima cat population compared to other species do not indicate low genetic diversity.

In a previous study using 12 microsatellite markers, the genetic diversity indexes in the Tsushima leopard cat were extremely low (*PID* and *PID-sib* were 2.1 × 10^−1^ and 4.5 × 10^−1^, respectively), so this microsatellite marker set could not perform identification of individuals [7]. In contrast, in this study, values of *PID* and *PID-sib* in the three genotyping methods were low enough that individual identification was thought to be possible. In addition, the high cumulative *PE* for the three methods indicated the possibility of identifying parentage in the Tsushima leopard cat. Therefore, the SNP marker set developed in this study can be applied to elucidate the animal life of the Tsushima leopard cat, including identification of individuals and estimation of kinship, and can be useful in conservation. In addition, in microsatellite analysis, it is necessary to have and share reference samples to compare microsatellite results among different laboratories. However, because SNP genotyping is easy to standardize among different laboratories, the SNPs discovered in this study can be useful for comparison of different research approaches (samples, machine, laboratory, and researcher).

Despite its high cost, for the Tsushima leopard cat, the best approach was to develop and use the draft genome. However, in non-model species, there is often no reference genome. Although both the *de_novo* and *ref_cat* methods were inferior to the *ref_TLC* version, they were considered useful for individual identification, parentage, and evaluation of genetic diversity. In the present study, the number of SNP markers was higher with the *ref_cat* method, which used the genome of the domestic cat, a closely related model species, as a reference, than in the *de_novo* method, but the *de_novo* method had higher genetic diversity indices. Mitochondrial DNA analysis estimated the divergence between *F. catus* and *P. bengalensis* to be 7.33 mya [49]. Considering these values and generation lengths for different species will lead to selecting better methods (de novo mapping or mapping to closely related species).

There were many SNPs that were excluded by deviations from the *HWE* (76.6% in *ref_TLC*, 78.5% in *ref_cat*, and 64.0% in *de_novo* were removed) (Table S2). Testing for the *HWE* in populations serves as a quality control step in genetic analysis and is a very common tool for detecting errors in sequencing and genotyping [50,51,52]. Similarly, in studies with a large number of markers, such as the Genome-Wide Association Study (GWAS), the *HWE* has been treated as a baseline for quality control [53,54]. However, various factors such as mutation, natural selection, nonrandom crossing, genetic drift, and gene flow can cause deviations from the *HWE* [55]. For example, deviations may occur in populations that have experienced inbreeding or have experienced recent bottlenecks. In populations with inbreeding experience, the value of *Ho* is smaller than the value of *He*, and in populations immediately after they undergo bottlenecking, the value of *He* is smaller than that of *Ho* (heterozygosity excess). Therefore, filtering by deviations from the *HWE* may blur the differences between significant deviations, such as those derived from evolutionary selection, and deviations due to genotyping errors. In this study, there was a large difference between mean *He* (0.313) and mean *Ho* (0.037) before *HWE* filtering. In addition to genotyping errors such as allele dropout, there may be many SNPs that misidentify a heterozygote as a homozygote due to low coverage. To derive a more accurate population structure, it is necessary to consider the effects of *HWE* filtering methods and to decrease typing errors by increasing coverage.

The best *K* values in the structure analysis were 1 to 3, according to the genotyping method and the *K* calculation method. In the analysis of population structure, Δ*K*, *Mean_LnP(K)*, or both are often used to calculate best *K* values. However, accurate estimation of *K* is difficult, and using Δ*K* and *Mean_LnP(K)* may overestimate or underestimate the value of *K* due to unbalanced sampling, low population differentiation, low marker information, hierarchical structure, and inbreeding [38,56]. Therefore, it is preferable to show plots of *Mean_LnP(K)* and Δ*K*, or to indicate structural bar plots of multiple *K* values [56]. It is also recommended that multiple methods of results be compared (e.g., structure analysis with BAPS or PCA) [56]. In this study, the results of the bar plots at *K* = 2 and *K* = 3 suggest that the individuals have a similar genetic composition and that all individuals belong to the same population, which in turn means that the Tsushima leopard cat has no subpopulations. This study also used the parsimony index [38], which differs from Δ*K* and *Mean_LnP(K)*, in calculating *K*. The parsimony method showed *K* = 1 for all three genotyping methods, and this value was the most suitable in the dataset of this study. It has been reported that the parsimony index can calculate *K* values that are more suitable than Δ*K* and *Mean_LnP(K)* under various conditions [38], and since these can be calculated from the output file from Structure as well as Δ*K* and *Mean_LnP(K)*, investigating *K* values together with Δ*K* and *Mean_LnP(K)* was considered effective.

In the PCoA results, data for most of the individuals were plotted in similar locations and no division within the population could be confirmed. However, the data for a few individuals (1–4) were located slightly apart from the others in the plot. Although the genetic differences were small, these individuals were thought to retain a slightly different genetic composition. It is thus possible to view these as high-priority individuals for captive breeding. In addition, increasing the sample size would clarify whether subpopulations exist or not. Breeding of the Tsushima leopard cat has been carried out in captive conservation with various genetic studies. However, because the genetic relationship between founders in captive populations is unknown, breeding plans are designed assuming that there is no kinship between the founders. Therefore, it is important to note the difference between genetic diversity based on pedigree and genetic diversity based on genetic analysis [57]. This is the case especially when the wild population is small, such that the difference between the two genetic diversities may be large [57]. The Tsushima leopard cat also has a small population size, so the genetic diversity it actually retains is likely to be lower than the genetic diversity based on pedigrees, and a more careful breeding plan is needed. In the future, the analysis of the captive population using these SNP markers can identify high-priority breeding founders and strains.

## 5. Conclusions

We identified more than one hundred SNP markers in the endangered Tsushima leopard cat by GRAS-Di analysis, which is one of the genome-wide analyses. These markers can be used in individual identification and parentage. We indicated that the Tsushima leopard cat population has no sub-population using these markers, this result can lead to the optimization of conservation efforts. Gras-Di analysis was useful for SNP discovery in endangered species. Our results will provide useful information for the conservation of the Tsushima leopard cat.

## Figures and Tables

**Table 1:** Genetic diversity indices of the Tsushima leopard cat in three genotyping methods.

		*N*	*Ne*	*Ho*	*He*
*ref_TLC*	Mean	38.823	1.104	0.084	0.088
	SE	0.153	0.008	0.007	0.006
*ref_cat*	Mean	39.000	1.099	0.076	0.083
	SE	0.150	0.009	0.007	0.006
*de_novo*	Mean	38.098	1.114	0.092	0.095
	SE	0.161	0.009	0.008	0.006

*N*: number of individuals, *Ne*: the number of effective alleles, *Ho*: observed heterozygosity, *He*: expected heterozygosity.

**Table 2:** Genetic diversity indices of the Tsushima leopard cat in three genotyping methods.

	*PID*	*PID-sib*	*PE*
*ref_TLC*	1.7 × 10^−11^	3.1 × 10^−5^	0.9987
*ref_cat*	1.3 × 10^−9^	2.6 × 10^−4^	0.9977
*de_novo*	1.9 × 10^−10^	1.0 × 10^−4^	0.9964

*PID*: cumulative probability of identity, *PID-sib*: cumulative *PID* among siblings, *PE*: cumulative probability of exclusion.

**Table 3:** The best *K* values in combinations three genotyping methods and three *K* estimators.

	*ref_TLC*	*ref_cat*	*de_novo*
Δ*K*	3	2	3
*Mean LnP(K)*	2	2	2
Parsimony	1	1	1

## Supplementary Materials

The following are available online at https://www.mdpi.com/2076-2615/10/8/1375/s1, Table S1: The numbers of loci and individuals at each filtering steps Table S2: Mean LnP(K), delta K, and parsimony index at each K in three genotyping methods.
